# The Hospital

**Published:** 1918-03-09

**Authors:** 


THE
### INSTITUTIONAL WORKER
**Being a Special Supplement to "The Hospital"**

### OUR BUREAU OF INFORMATION.

### Rules for Correspondents.

1. Every letter must be accompanied by the coupon to be cut from the back cover (inside page) of THE HOSPITAL, current issue, and must contain the name and address of the correspondent with pseudonym for publication if desired. Replies by post cannot be given save under exceptional circumstances at the Editor's discretion.

2. Letters from Approved Homes in reply to special needs published in the Bureau should state terms and full particulars, and be sent prepaid under cover to the Editor of the Bureau with name written across coupon for identification.

3. Proprietors of Homes which have not yet been entered on the List of Approved Homes, but have spare accommodation likely to suit special needs, are invited to write for an application form for registration. The fee for registration, which includes two announcements of the Home in the Bureau and other privileges, is 10s.

4. All communications to be addressed to the Editor of THE HOSPITAL, 28 Southampton Street, Strand, London, W.C. 2, and marked "Bureau of Information."

### INSTITUTION FACTS AND FIGURES.

### Crockery Washing in a Nurses' Home.

**Answer to January Question.**

**By "Matron."**

THE question was:

**Describe the cleanest, quickest, and most careful method of washing and drying crockery in a nurses' home to accommodate 180 persons.**

The washing-up of crockery for so many persons should be done in a special pantry, fitted with a sink, hot and cold water, draining-board, plate-rack, and a large table.

Two persons should be responsible for the work, and be provided with plenty of clean glass- and tea-cloths, and they must wear large aprons or overalls. Two large bowls, or small baths, will be necessary to wash the articles; one should contain soap-powder, or soda, and hot water, the other plain hot water.

When the crockery is brought down to be washed, one worker can get ready the bowls of hot water, while the other sorts the articles, putting away salt-cellars, serviettes, and trays, and scraping any scraps or grease from the plates, placing the spoons and forks, knives and plates in separate piles.

If there are any glasses, these should be washed first in the bowl with the soap-powder or soda, then rinsed in the other bowl of plain hot water, and dried on a clean glass-cloth. The silver may be washed next, in the same manner; then any cups and saucers, and lastly the knives and plates. The water should be changed frequently, and large plates can be washed with a plate-mop in soapy, then plain, water, and put in the plate-rack to dry.

It may be necessary to put some of the articles away in the cupboards before washing the rest. If the silver is rubbed with a wash-leather before putting it away, it will keep bright and require cleaning less often.

### How to Sterilise Rubber Gloves.

**Answer to February Question.**

**By "A. M."**

THE question was:

**Describe the best method of sterilising rubber gloves, to be used during operations, to ensure their being perfectly sterilised with the minimum amount of damage to the rubber.**

There are two satisfactory methods of rendering rubber gloves aseptic—viz., wet and dry sterilisation.

(1) STERILISATION BY DRY HEAT.

Dry, patch, and well powder gloves, and put each pair into a separate bag or towel, together with a small packet of powder (French chalk). To ensure each person getting a suitable size of gloves, the bags or towels should be marked with names of all theatre assistants.

Place all packages in a drum, which is then subjected to steam pressure, 10 lb. to the square inch, for half an hour.

At the time of operation the hands, after the usual amount of preparation, are dried on a sterile towel, and a small quantity of powder out of the sterile packet lightly rubbed into hands, when the gloves can be drawn on quite easily, particularly if the wrists of the gloves have been previously turned up over the palm. A sterile bowl or tray should be provided as a receptacle for the second glove whilst the first is being put on, or, if the bag is kept aseptic, it may remain in the drum.

(2) STERILISATION BY BOILING.

Put each pair of gloves in separate bags or towels as before, place in boiling water, and allow to boil for twenty minutes; take out with forceps and place in a sterile bowl. After the hands are prepared, ethereal soap is generally used to enable the gloves to be drawn on easily. This method of sterilisation is especially suitable for the cleansing of gloves after use in septic cases, and for such cases it is advisable to keep a separate set of gloves.

THE TWO METHODS COMPARED.

The disadvantages of (2) are:

(a) Much discomfort and consequent "chapping" are caused by the soap-and-water trickling down the arms and the hands becoming very sodden and saturated with moisture.

(b) The rubber thickens and becomes rotten and perishes more quickly than by the dry method.

(c) The gloves frequently split at the wrist by the vigorous pulling on they sometimes require.

The advantages of the dry method (1) are:

(a) The hands are kept quite dry as long as the gloves are worn, and therefore are much more comfortable.

(b) The sterilising room is kept cleaner and drier (many a "pro." slips and falls on a floor which is flooded with soap-and-water due to the putting on of gloves sterilised by the wet method).

(c) The gloves slip on so easily that there is very little possibility of them being torn at the wrist.

(d) The rubber remains thin and pliable, and is supposed to last much longer.

The dry method is generally considered infinitely preferable to the wet by surgeons who have tried both.

### MARCH QUESTIONS.

### (1) The Admission of Patients.

Is it an advantage to admit patients to small hospitals (under 50 beds) by subscribers' letters, or is the system of admission on a doctor's order only preferable.

### (2) Repairs and Renewals.

Describe the system adopted at your institution for dealing with repairs, including (a) the arrangement in force for the periodical examination of the structure, and (b) the method of collecting, recording, and disposing of repair requisitions.

#### RULES.

The following rules must be observed:—

1. Contributions must be written on one side of the paper. Brevity and terseness are desirable features. The MSS. must bear the name and address of the sender and be accompanied by coupon to be cut from the back cover (inside page) of the current issue of THE HOSPITAL. A pseudonym must be chosen if the name is not to be published.

2. Contributions must be addressed to the Editor of THE HOSPITAL, Institutional Worker Supplement, 28 & 29 Southampton Street, Strand, London, W.C. 2, should reach him before the end of the current month, and be marked in the left-hand corner "Facts and Figures."

**A minimum payment of Five Shillings will be made for each published answer.**

### QUESTIONS INVITED.

IN connection with our Question Box, we offer every month five shillings each for the two best questions which are sent in for consideration. The questions may be on any institutional subject and concern any department, from the secretary's to the porter's, or the matron's to the domestic staff; the one essential is that they must be practical and deal with points that have a definite relation to hospital or institutional administration, upkeep, finance, or management. Points relating to artificers' work, housekeeping, the laundry, out-patients, and other practical matters will be welcome. It is hoped that thus, when difficult or doubtful points arise in the course of their work, institutional workers will be encouraged to put them in the form of a question and send them to the Editor, THE HOSPITAL Bureau of Information, 28 & 29 Southampton Street, Strand, W.C. 2, marked "Question Box."

The best questions will be published in due course and our readers will be given the opportunity of answering them. Thus all institutional workers may be able to cooperate with the view of helping the work and smoothing the difficulties of each other. In short, we want the Question Box of the *Institutional Worker* to be freely used by every worker habitually.

### ENQUIRIES AND ANSWERS.

### *THE SICK AND IN NEED.*

### Care of Mental Cases.

A LICENCE is necessary for the reception of certified lunatics into a home, and new licences are very difficult to obtain. With the exception of the Counties of London and Middlesex, the Cities of London and Westminster, and certain parishes within a radius of seven miles which come within the jurisdiction of the Lunacy Commissioners, the licensing jurisdiction is exercised by the Justices of every county and quarter sessions borough. There are no statutory regulations regarding the reception of mild mental cases which are uncertified, for such cases do not come within the scope of the Lunacy Act.—R. M.

### Male Epileptic.

THE Co-operative Sanatoria, Ltd., Mill House, Billericay, Essex, might accept the case in question. The charges in pre-war days were from 16s. to £2 2s. weekly. This sanatorium admits male epileptics of the middle classes, and also mental patients not requiring asylum treatment. Application should be made to the Secretary for admission.—TONY.

### *EMPLOYMENT AND TRAINING.*

### Training for Social Work.

WE suggest that you write to the Secretary of the Charity Organisation Society, Denison House, Vauxhall Bridge Road, London, S.W., for full details regarding a course of training arranged by the Bedford College for Women (University of London) and the Charity Organisation Society. The course includes a year's theoretical work at Bedford College, divided into three terms, and practical work in an office of the Charity Organisation Society, where an insight is given into the work of infant welfare centres, tuberculosis dispensaries, and the Invalid Children's Aid Association.—MORRIS.

### Training at Thirty-Three.

AMONG other institutions of over 100 beds the Royal Hants County Hospital, Winchester, receives probationers up to thirty-three years of age, and makes arrangements for their training in massage and electrical work. There is a private nursing staff attached, on which nurses are required to serve in their fourth year.—OXFORD.

### *THE HOUSEKEEPERS' DEPARTMENT.*

### Corned Beef and Vegetable Pie.

LINE a pie-dish with some mashed potato, add a layer of corned beef, and over this pour some gravy (gravy made with Bisto is quite good); next add some chopped onion and carrot, partly cooked, and seasoning to taste. Cover this with more mashed potato. Bake until nicely browned—about half an hour.—RATIONS.

### Potato and Apple Sweet.

THE following makes quite a nice vegetarian dish: Boil 1½ lb. of potatoes, and mash while hot; add two tablespoonfuls of milk, and two of sugar, a piece of margarine the size of a large walnut, and add a few drops of vanilla flavouring if desired. Line a small pie-dish with this mixture, and fill up with 1½ lb. of stewed apples (weighing 1½ lb. before cooking), and cover with the remaining potato mixture. Bake until nice and brown. This makes sufficient for about six people.—MAXIM.

### A Steak Substitute.

THE following formula for steak substitute was recently suggested by a food expert. It contains all the necessary food constituents which enable it to take the place of steak. You will require one egg, 1 oz. Bovril, one tablespoonful flour, five tablespoonfuls of breadcrumbs, and ½ oz. of dripping or butter. Break the egg in a basin, add the Bovril, then the flour (which should previously have been mixed with the dripping or butter), and with the breadcrumbs make the whole into a stiff paste, dip it into the flour, and fry a golden brown in hot fat. The cost of these croquettes would be 10d., assuming the egg to cost 4½d., and would be sufficient for two persons. These croquettes are very appetising, and are rich in nitrogen and all other food constituents essential to proper nourishment, as well as being particularly easy to digest.—HOUSEKEEPER.

### *MISCELLANEOUS.*

### Selling Waste Paper.

WE suggest that you dispose of your waste paper to the paper mills. If this is not possible in your district, the Paper Mills Supply Company, waste-paper contractors, 9A Penarth Road, Cardiff, buy all classes of waste paper, and will give you quotations if you will write to the secretary.—HOSPITAL SECRETARY.

### New Coolidge Tube.

THE new fine-focus Coolidge Tube with bulb 3¾ in. in diameter is suitable for relatively small energy outputs only, ranging from 25 milliamperes over a 2-in. gap to 8 milliamperes over a 6-in. gap. Among the special advantages is the small diameter of the bulb, which permits of the closer approach of the anode to the diaphragm of the tube-box, resulting in an improvement in the definition of shadows. The tube is especially useful for lung and stomach examinations and for dental radiography.—X. Y. Z.

